# Reply to Laby, D.M.; Appelbaum, L.G. Comment on “Nascimento et al. Citations Network Analysis of Vision and Sport. *Int. J. Environ. Res. Public Health* 2020, *17*, 7574”

**DOI:** 10.3390/ijerph18126521

**Published:** 2021-06-17

**Authors:** Henrique Nascimento, Clara Martinez-Perez, Cristina Alvarez-Peregrina, Miguel Ángel Sánchez-Tena

**Affiliations:** 1 ISEC Lisboa, Instituto de Educação e Ciência de Lisboa, 1750-179 Lisboa, Portugal; henrique.nascimento@iseclisboa.pt (H.N.); masancheztena@ucm.es (M.Á.S.-T.); 2 Faculty of Biomedical and Health Science, Universidad Europea de Madrid, 28670 Madrid, Spain; cristina.alvarez@universidadeuropea.es; 3 Department of Optometry and Vision, Faculty of Optics and Optometry, Universidad Complutense de Madrid, 28670 Madrid, Spain

## Introduction

In response to a comment, in this study [1] we have performed a citation network analysis, not a bibliometric analysis. Carrying out this type of analysis at some point in time will permit knowledge of the existing bibliography networks, which establish the citations in academic publications that represent a specialty and a scientific community. In turn, the structures and characteristics of said specialty and community can be studied. By making comparisons between time periods, the historical development of the specialty and the community can be modeled. In the analysis of dating networks, a set of objects (documents, authors, journals, or groups of them) that represent a research field is selected. The strengths of the interrelationships (or levels of connection) between these data are analyzed using various scores that come from citation counts, structures, and characteristics of the corresponding research fields and academic communities. To reveal the structures underlying these relationships, multivariate statistical analyses are often applied using citation scores as measures of similarity. For this reason, network visualization tools are also frequently used to produce visual maps of these relationships [2].

Depending on the units of analysis (documents or groups of them by authors, journals, research fields, nations, etc.) and the thresholds of citation scores, both macrostructures (general maps of the entire scientific effort with each node in the La network representing a discipline) and microstructures (structures of a single specialty with each node of the network representing a single document) of science can be mapped and studied, allowing the user to access general descriptions of the fields of investigation, as well as exploring the underlying structures [3].

There are three types of commonly used citation-based measures of the strength of the interrelation between two objects [3]:

- Interleaved citation count—the number of times two objects have cited each other;
- Co-citation counts—the number of documents that have cited two objects together;
- Bibliographic coupling frequencies (BCF)—the number of cited references that have two objects in common.

Therefore, we agree with the given comments. However, it was not the analysis that was carried out in our study [1], and that is why the data cited in the comment were not obtained.
